# Role of Long Intergenic Nonprotein-Coding RNA 00511 in Nod-Like Receptor Protein Pyrin Domain 3-Induced Chondrocyte Pyroptosis via the MicroRNA-9-5p/FUT1 Axis

**DOI:** 10.4014/jmb.2312.12014

**Published:** 2024-04-15

**Authors:** Tianjun Zhai, Zengqiao Zhang, Xiaoshen Hu, Dongyi He, Wei Feng

**Affiliations:** 1 School of Rehabilitation Science, Shanghai University of Traditional Chinese Medicine, Shanghai 201203, P.R. China; 2 Shanghai University of Traditional Chinese Medicine Rehabilitation Institute, Shanghai 201203, P.R. China; 3 Tuina Department of Yueyang Hospital of Integrated Traditional Chinese and Western Medicine Affiliated to Shanghai University of Traditional Chinese Medicine, Shanghai 200083, P.R. China; 4 School of Health Preservation and Rehabilitation, Chengdu University of Traditional Chinese Medicine, Chengdu 610075, P.R. China; 5 Rheumatoid Internal Medicine in Shanghai Guanghua Hospital of Integrated Traditional Chinese and Western Medicine, Shanghai 200052, P.R. China; 6 The Second Rehabilitation Hospital of Shanghai, Shanghai 200441, P.R. China

**Keywords:** Osteoarthritis, chondrocyte, inflammatory injury, long intergenic nonprotein coding RNA 00511, microRNA-9-5p, FUT1

## Abstract

This study aimed to determine the function of LINC00511 in Nod-Like Receptor Pyrin Domain 3 inflammasome-mediated chondrocyte pyroptosis via the regulation of miR-9-5p and FUT 1. Chondrocyte inflammatory injury was induced by treating chondrocytes with LPS. Afterwards, the levels of IL-1β and IL-18, the expression of NLRP3, ASC, Caspase-1, and GSDMD, cell viability, and LDH activity in chondrocytes were assessed. LINC00511 expression in LPS-treated chondrocytes was detected, and LINC00511 was subsequently silenced to analyse its role in chondrocyte pyroptosis. The subcellular localization of LINC00511 was predicted and verified. Furthermore, the binding relationships between LINC00511 and miR-9-5p and between miR-9-5p and FUT1 were validated. LINC00511 regulated NLRP3 inflammasome-mediated chondrocyte pyroptosis through the miR-9-5p/FUT1 axis. LPS-treated ATDC5 cells exhibited elevated levels of inflammatory injury; increased levels of NLRP3, ASC, Caspase-1, and GSDMD; reduced cell viability; increased LDH activity; and increased LINC00511 expression, while LINC00511 silencing inhibited the NLRP3 inflammasome to restrict LPS-induced chondrocyte pyroptosis. Next, LINC00511 sponged miR-9-5p, which targeted FUT1. Silencing LINC00511 suppressed FUT1 by upregulating miR-9-5p. Additionally, downregulation of miR-9-5p or overexpression of FUT1 neutralized the suppressive effect of LINC00511 knockdown on LPS-induced chondrocyte pyroptosis. Silencing LINC00511 inhibited the NLRP3 inflammasome to quench Caspase-1-dependent chondrocyte pyroptosis in OA by promoting miR-9-5p and downregulating FUT1.

## Introduction

As one of the most common types of arthritis, osteoarthritis (OA) is characterized by the proliferation of subchondral bones and degeneration of articular cartilage [1]. OA is a serious and complicated disorder resulting from a series of factors, such as uric acid, heredity, smoking, body weight, and cardiovascular disease [2, 3]. Currently, the occurrence and disability rates of chronic OA are increasing, which could lead to debilitating living standards, deficient functions, medical expenditures, and social burdens [4]. Currently, feasible management strategies for OA include physical exercise, intra-articular treatment, and the administration of nonsteroidal anti-inflammatory drugs [3]. Mechanically, intensive and persistent pyroptosis or inflammation in soft tissues might induce augmented anguish or hypersensitivity when subjected to injurious stimulation and eventually unleash cytokines to strengthen pain from OA [5]. Unfortunately, inflammatory injury, which is similar to rheumatoid synovial infiltration, is the major indication of OA, making it difficult to histologically distinguish these two kinds of disease [6]. Therefore, this paper is expected to identify potential strategies for OA alleviation.

Long noncoding RNAs (lncRNAs) influence a wide range of gene biological activities and are responsible for the incidence of different human pathological processes, including OA [7]. As a member of the lncRNA family, LINC00511 functions as a tumour propeller in diverse carcinomas, including hepatocellular carcinoma and cervical cancer, leading to active lymphatic metastasis, worsening clinical consequences, promoting cellular processes, suppressing apoptosis, and increasing drug resistance [8, 9]. According to other reports lncRNA MALAT1 is highly expressed in chondrocytes after LPS treatment [10], and MCM3AP-AS1 is also highly expressed [11], which suggests that there may be a variety of lncRNAs whose expression levels change in chondrocytes after LPS treatment. Relatively speaking, the effect of LINC00511 on noncancerous diseases has been insufficiently studied, but as Zhang and his colleagues reported, LINC00511 impedes chondrocyte growth and promotes extracellular matrix (ECM) and cell death in OA [12]. Therefore, LINC00511 has detrimental effects on chondrocyte formation and development in OA. Since it has been repeatedly reported that LINC00511 principally affects cancer progression by serving as a competing endogenous RNA (ceRNA) via the lncRNA/microRNA (miR)/mRNA axis [13, 14], we hypothesize that LINC00511 could also mediate OA severity through the ceRNA network.

miRs are necessary players and significant predictors of cartilage homeostasis and chondrocyte growth in OA, as their absence exacerbates osteal ill-growth and degradation, while their participation contributes to the modulation of molecular senescence, cell death, and cellular self-renewal of chondrocytes [15]. miR-9-5p overexpression suppresses apoptosis, accelerates chondrocyte expansion and cartilage reconstruction, and attenuates OA pathological severity [16]. Similar to LINC00511, miR-9-5p has not been reported to be related to chondrocyte pyroptosis in the literature; therefore, we chose this miRNA for our study. In addition, multiple studies have shown that miR-9-5p functions as a sponge of lncRNAs to modulate different human diseases [17, 18], indicating the feasibility of using miR-9-5p as a ceRNA network. Fucosyltransferase (FUT) 1 promotes endothelial cell mobility and exacerbates angiogenesis in rheumatoid arthritis [19]. FUT1 is overexpressed in OA-and inflammation-induced chondrocytes, reduces chondrocyte viability, and exacerbates ECM injury [20]. Taken together, these findings suggest that LINC00511 might play a role in OA chondrocyte pyroptosis by regulating the miR-9-5p/FUT1 axis.

## Materials and Methods

### Cell Cultivation and Treatment

Mouse chondrocytes (ATDC5) (American Type Culture Collection, USA) were cultured in Dulbeccós modified Eaglés medium/Ham's nutrient mixture F12 (DMEM/F12, Gibco, Life Technologies, USA) supplemented with 10% (v/v) foetal bovine serum (Gibco, Life Technologies) and 1% (v/v) penicillin‒streptomycin-glutamine (100×, Gibco, Life Technologies) in a 5% CO_2_, 37°C environment [7].

Lipopolysaccharide (LPS, Sigma‒Aldrich, Merck KGaA, Germany) was diluted in ultrapure water (5 mg/ml). LPS solution diluted to 1 μg/ml in serum-free DMEM/F12 was applied to ATDC5 cells for 12 h, and the cells were used for further experiments [7]. Cells without LPS treatment were used as the control group.

### Cell Transfection

The miR-9-5p mimic, miR-9-5p inhibitor, small interference (si)-LINC00511, overexpression (oe)-FUT1, and negative control (NC) (all from Shanghai GenePharma Co., Ltd., China) were transfected into ATDC5 cells following the instructions of Lipofectamine 2000 (Invitrogen Inc., USA). After 48 h of transfection, the ATDC5 cells were subjected to further steps.

### Quantitative Real-Time Polymerase Chain Reaction (qRT‒PCR)

An RNeasy Mini kit (Qiagen, USA) was used to reverse transcribe total RNA to cDNA using reverse transcription kits (RR047A, Takara Bio Inc., Japan). miR was assessed and reverse transcribed to cDNA using miR First Strand cDNA Synthesis (Tailing Reaction) kits (B532451-0020, Shanghai Sangon Biotech Co., Ltd., China). SYBR Premix Ex TaqTM II (Perfect RT) kits (DRR081, Takara) and a qRT‒PCR instrument (ABI7500, ABI, Inc., USA) were utilized to carry out qRT‒PCR. The product was amplified by a two-step process and divided into initial denaturation at 95°C for 30 s and PCR, which included 40 cycles of 95°C for 5 s and at 60°C for 34 s, with 3 duplicate wells for each sample. The primer sequences (Table 1) used were designed by Sangon Biotech. The Ct value of all wells was evaluated, with glyceraldehyde-3-phosphate dehydrogenase (GAPDH) or U6 serving as the internal reference. The 2^-ΔΔCT^ method was used to measure the relative expression of genes. The formula was as follows: ΔΔCT= ΔCt experimental group target gene–ΔCt experimental group housekeeping gene)-(ΔCt = Ct NC group target gene–Ct NC group housekeeping gene).

### Interleukin (IL)-1β and IL-18 Expression Detected via Enzyme-Linked Immunosorbent Assay (ELISA)

Cells subjected to transfection and LPS treatment were collected for culture of the supernatant. After that, IL-1β and IL-18 released into the medium were analysed via ELISA kits (R&D Systems Inc., USA) following the manufacturers’ instructions. Next, the optical density at 450 nm was evaluated. Then, the levels of IL-1β and IL-18 were determined according to standard curves [21].

### Expression of the Nod-Like Receptor Pyrin Domain 3 (NLRP3) Inflammasome and FUT1 Assessed by Western Blot Analysis

Cells were lysed using an enhanced RIPA lysis buffer (Boster Biological Technology Co., Ltd., China) containing protease inhibitors. Subsequently, the protein concentration was evaluated via bicinchoninic acid protein quantification kits (Boster). Next, proteins were separated by 10% sodium dodecyl sulfate (SDS) polyacrylamide gel electrophoresis before being transferred to polyvinylidene fluoride membranes. Then, proteins were blocked with 5% bovine serum albumin for 2 h to block nonspecific binding and then cultured with diluted primary antibodies against Caspase-1 (1:1000, 4199, Cell Signaling Technology, USA), Gasdermin D (GSDMD-N)(ab215203, 1:1000, Abcam Inc., USA), NLRP3 (ab214185, 1:1000, Abcam), apoptosis-associated speck-like protein (ASC) (1:100, sc-514414, Santa Cruz), and GAPDH (ab9485, 1:2500, Abcam) at 4°C overnight. Afterwards, the membranes were incubated with horseradish peroxidase-conjugated goat anti-rabbit immunoglobulin G (IgG, ab205718, 1:2000, Abcam) or goat anti-mouse IgG (ab205719, 1:2000, Abcam) secondary antibodies for 1 h. Then, the membranes were developed with an enhanced chemiluminescence reagent (Millipore Corp., USA). Image-Pro Plus 6.0 (Media Cybernetics, USA) was used to measure the grey value of each band in the western blot images, with GAPDH serving as an internal reference. All procedures were carried out 3 times.

### Caspase-1 Levels Detected by Immunofluorescence [22]

Cells in different groups were inoculated into 6-well cover glasses coated with polyethyleneimine (0.04% v/v, Sigma‒Aldrich) and cultured in a 37°C humid incubator with 5% CO_2_ and 95% air. When the cells reached 80-90%confluence, they were used for experiments. First, the cover glasses were fixed with 4% paraformaldehyde and then cultured with the mouse monoclonal antibody Caspase-1 (1:50, sc-392736, Santa Cruz) in a humid and dark environment at 4°C for 1 h. Then, the cover glasses were cleaned with 0.1% Triton-X and 1×phosphate buffer saline (PBS) (PBST) 3 times (5 min/wash) and then cultured with goat anti-mouse IgG (Alexa Fluor 488, ab150113, 1:200, Abcam) in a humid and dark environment at 4°C for 1 h. Subsequently, the cover glasses were subjected to another 3 washes in 0.1% Triton-X and 1×PBS (PBST) (5 min/time) and cultivated with DAPI for 10 min for nuclear counterstaining. Consequently, cover glasses were sealed on glass slides with Vectashield fluorescent installation medium (H-1000, Shanghai Sangerbio Biotechnology Co., Ltd.). Images were captured using a fluorescence microscope (Olympus Optical Co., Ltd., Japan).

### Cell Viability Measurement [22]

Chondrocyte viability was measured by Cell Titer Glo Luminescent Cell Viability Assay kits (G7570, Promega Corp., China). Cells (1 × 10^5^) were seeded into 6-well plates with normal growth medium and harvested for further experiments when cell confluence reached 80-90%. Before the cells were collected, the reagents were prepared following the manufacturers’ instructions. Next, cells (50 μl) from different groups were inoculated into black-walled 96-well plates containing an equal amount of final substrate solution from the CellTiter-Glo Luminescent Cell Viability Assay, which was mixed and cultivated with cells for 10 min for detection with the assistance of the instructions of a plate-reading luminometer (Varioskan, Thermo Scientific Pierce, USA).

### Lactate Dehydrogenase (LDH) Release Assay [1]

LPS-induced cytotoxicity was determined by LDH release assay kits (Beyotime Biotechnology Co. Ltd., China). Chondrocytes treated with LPS were seeded into 96-well plates and cultivated in a 37°C incubator with 5% CO_2_ for 24 h. Then, the cell medium was collected for LDH activity assessment according to the manufacturer’s instructions.

### LINC00511 Subcellular Localization by Fractionation of Nuclear and Cytoplasmic RNA

Chondrocytes were resuspended in hypotonic buffer A (10 mM HEPES (pH 7.5), 0.5 mM dithiothreitol (DTT), 10 mM KCl, and 1.5 mM MgCl_2_) supplemented with protease inhibitor cocktail and RNase inhibitor (N8080119, Thermo Fisher Scientific). The cells were incubated on ice for 10 min and centrifuged at 1,000 ×*g* at 4°C for 10 min. The cytoplasm was obtained as the supernatant was further centrifuged at 15,000 ×*g* for 15 min. The precipitates were rinsed twice with hypotonic buffer, resuspended in Hypotonic buffer B (10 mM HEPES (pH 7.5), 10 mM KCl, 1.5 mM MgCl_2_, 0.5 mM DTT and 0.5% Nonidet P-40), cultured at 4°C for 30 min, with gentle rotation and centrifuged at 6,000 ×*g* at 4°C for 10 min. Subsequently, the precipitates were cleaned once with hypotonic buffer, resuspended in RIPA buffer (50 mM Tris HCl (pH 7.5), 1,500 mM KCl, 1% Nonidet P-40, 0.5% sodium deoxycholate, 0.1% SDS, 1 mM ethylene diamine tetraacetic acid (pH 8.0)), cultivated at 4°C for 30 min, gently rotated and then centrifuged at 15,000 ×*g* for 20 min. The supernatant was identified as the nucleus [23].

### Dual-Luciferase Reporter Gene Assay

The binding sites of LINC00511 and miR-9-5p, and miR-9-5p and FUT1 were analysed via the Starbase database (http://starbase.sysu.edu.cn/index.php). Then, wild-type (WT) and mutant (MUT) fragments of LINC00511 and FUT1 were inserted into pMIR reporter plasmids (Beijing Huayueyang Biotechnology, China). Next, luciferase reporter plasmids were cotransfected with mimic NC or miR-9-5p mimic into HEK293T cells (Shanghai Beinuo Biotechnology, China). After 48 h of transfection, the chondrocytes were collected and lysed using luciferase assay kits (K801-200, BioVision Inc., USA) for the assessment of luciferase activity.

### RNA Immunoprecipitation (RIP)

RIP kits (Millipore) were used to analyse the interactions between miR-9-5p and FUT1 and between LINC00511 and miR-9-5p. Chondrocytes were washed with precooled PBS to extract the supernatant. Then, the chondrocytes were lysed with equal amounts of ice-cold RIPA lysis buffer (P0013B, Beyotime) and centrifuged for 10 min at 4°C to extract the supernatant. Part of the chondrocyte extract solution was separated as input, and the other part was used for culture with antibodies for coprecipitation. RNA was extracted from the samples by proteinase K and subsequently subjected to LINC00511, miR-9-5p, and FUT1 expression detection by qRT‒PCR. The antibodies (Abcam) applied in RIP included Argonaute 2 (1:100, ab109489) (mixed for 30 min) and rabbit anti-IgG (1:100, ab109489) (as the NC). Every procedure was performed 3 times.

### Statistical Analysis

The data were analysed by SPSS 21.0 (IBM Corp., USA), and the images were graphed using GraphPad Prism 8.0 software (GraphPad Software, USA). The data are expressed as the mean ± standard deviation. The data were normally distributed. Comparisons between two groups were performed by t tests, and comparisons among among multiple groups were performed by one-way or two-way analysis of variance (ANOVA); pairwise comparisons after ANOVA were performed via Tukey's post hoc multiple comparisons test. A difference was considered statistically significant at a *p* value < 0.05.

## Results

### LPS Activates the NLRP3 Inflammasome to Induce Chondrocyte Pyroptosis and Upregulate LINC00511 Expression

**Fig. 1:**
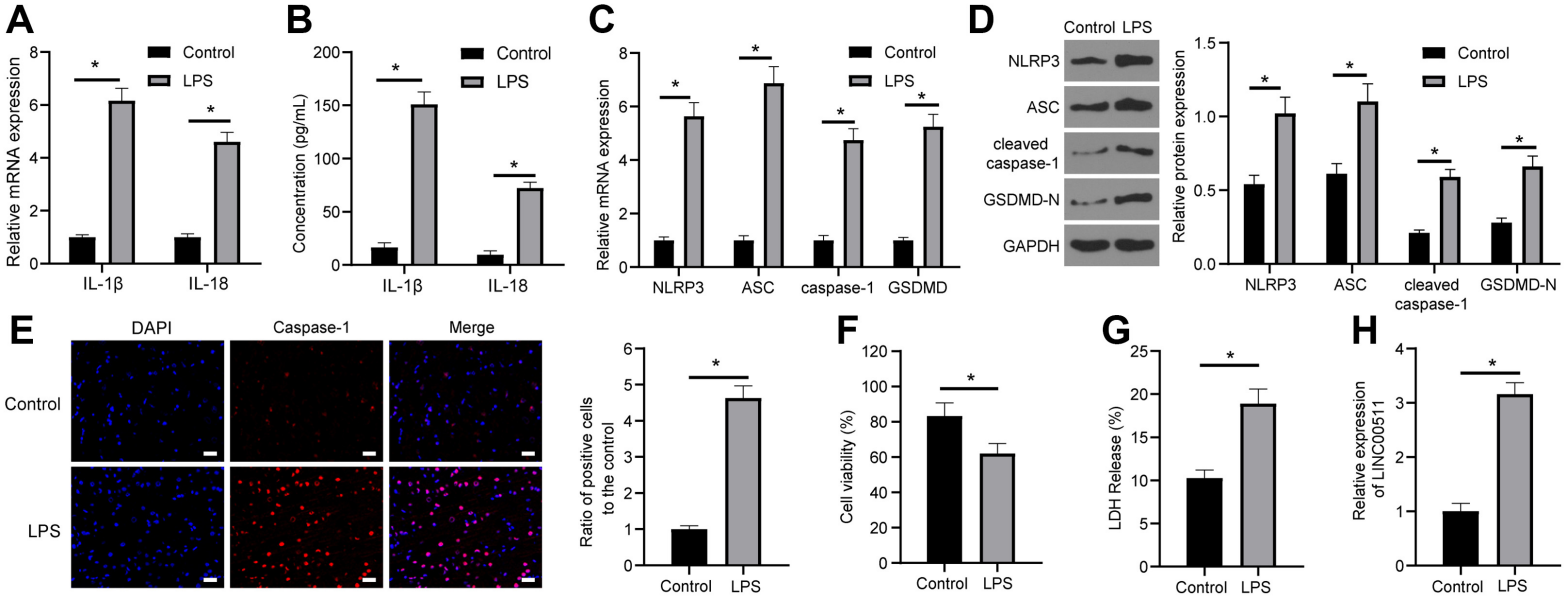
LPS activates the NLRP3 inflammasome to induce caspase-1-induced chondrocyte pyroptosis and upregulates LINC00511 expression. ATDC5 cells were treated with LPS to induce chondrocyte inflammatory injury. Cells without LPS treatment were used as the control group. A and B, Levels of IL-1β and IL-18 verified by qRT‒PCR (**A**) and ELISA (**B**). (**C**) and (**D**), The levels of NLRP3, ASC, Caspase-1, and GSDMD were tested using qRT‒PCR (**C**) and western blot analysis (**D**). (**E**) Caspase-1 levels were assessed by dual-luciferase reporter gene assays at 400×; scale bar = 25 μm. (**F**) Cell viability was analysed by Cell Titer Glo Luminescent Cell Viability Assay kits. (**G**) LDH viability measurement. (**H**) LINC00511 expression was tested through qRT‒PCR. Three repeated tests were performed. The results are presented as the mean ± standard deviation. Two-way ANOVA was used to analyse the data in Panels A, B, C, and D. Tukey's multiple comparisons test was used for the post hoc test. Independent t tests were used to analyse the data in Panels E, F, G, and H. *p < 0.05.

To further investigate the molecular interaction of pyroptosis in OA, chondrocytes (ATDC5 cells) were initially treated with LPS to induce inflammatory injury in chondrocytes. Then, the levels of IL-1β and IL-18 were evaluated by qRT‒PCR and ELISA, which revealed that compared with those in the control group, the levels of IL-1β and IL-18 were elevated in the LPS group (all *p* < 0.05) (Fig. 1A and 1B). In addition, the levels of pyroptosis-related proteins (NLRP3, ASC, Caspase-1, and GSDMD) were verified, and compared with those in the control group, the LPS group exhibited elevated levels of pyroptosis-related proteins (all *p* < 0.05) (Fig. 1C and 1D). On the other hand, according to the results of dual-luciferase reporter gene assays, Caspase-1 was upregulated in the LPS group (*p* < 0.05) (Fig. 1E). Moreover, compared with the control group, the LPS group exhibited decreased chondrocyte viability (*p* < 0.05) (Fig. 1F) and an increased LDH release rate (*p* < 0.05) (Fig. 1G). These findings illustrated that LPS activated the NLRP3 inflammasome to induce Caspase-1-dependent chondrocyte pyroptosis.

To verify the role of LINC00511 in OA via its mediation of chondrocyte pyroptosis, LINC00511 expression in LPS-treated ATDC5 cells was tested through qRT‒PCR, which revealed that LINC00511 expression was greater in the LPS group than in the control group (*p* < 0.05) (Fig. 1H), illustrating that LINC00511 is importantly involved in LPS-induced chondrocyte pyroptosis.

### LINC00511 Knockdown Inhibits the NLRP3 Inflammasome to Limit LPS-Induced Chondrocyte Pyroptosis

**Fig. 2:**
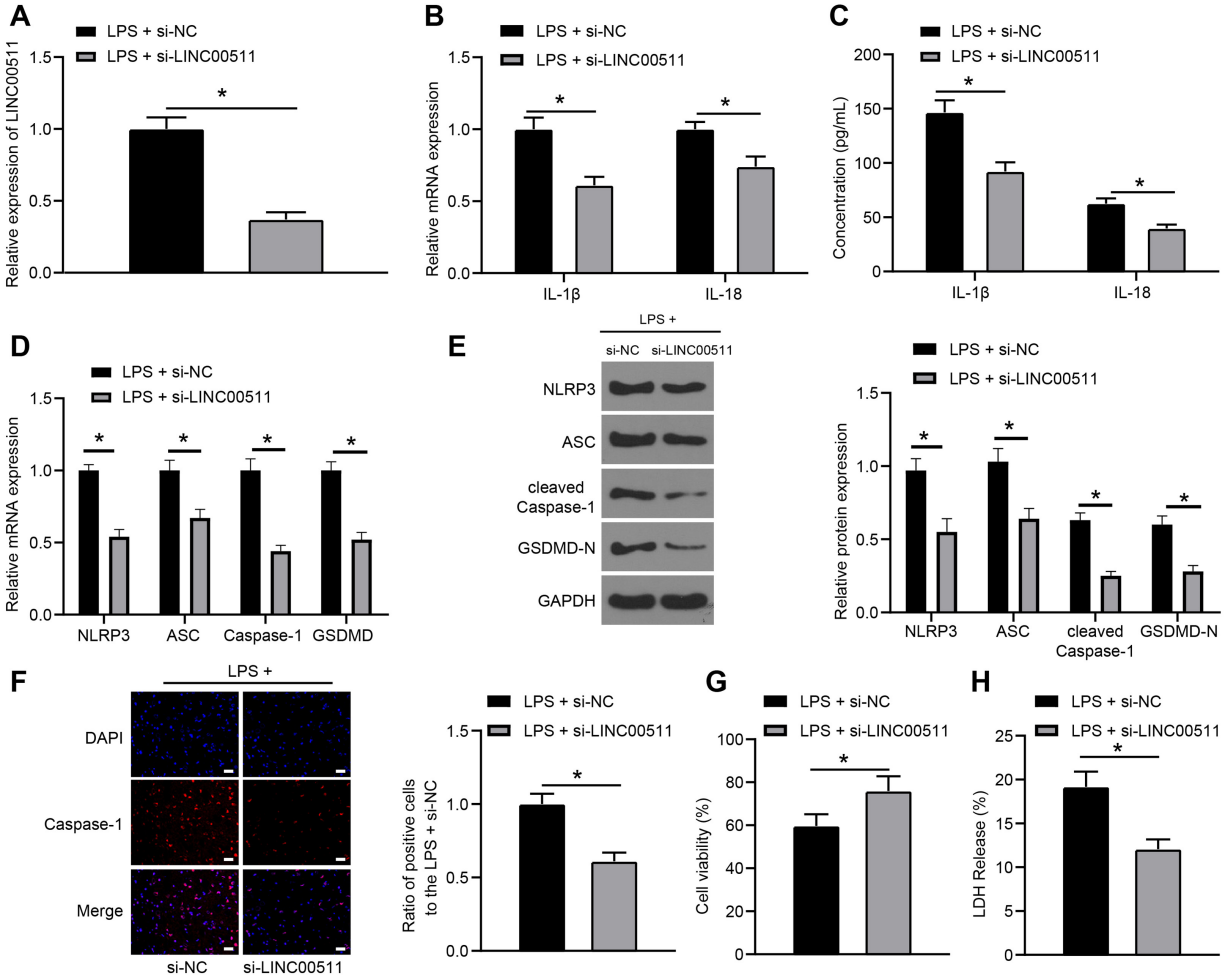
LINC00511 knockdown inhibits the NLRP3 inflammasome to limit LPS-induced chondrocyte pyroptosis. si-NC and si-LINC00511 were transfected into LPS-treated ATDC5 cells. (**A**) LINC00511 expression was examined via qRT‒PCR. B and C, Levels of IL-1β and IL-18 determined by qRT‒PCR (**B**) and ELISA (**C**). D and E, The levels of NLRP3, ASC, Caspase-1, and GSDMD were detected via qRT‒PCR (**D**) and western blot analysis (**E**). (**F**) Caspase-1 levels were assessed by dual-luciferase reporter gene assays at 400×; scale bar = 25 μm. (**G**) Cell viability was tested with Cell Titer Glo Luminescent Cell Viability Assay kits. (**H**) LDH viability measurement. Three independent repeated tests were conducted. The results are presented as the mean ± standard deviation. Independent t tests were used to analyse the data in Panels A, F, G, and H. Two-way ANOVA was used to analyse the data in Panels B, C, D, and E. Tukey's multiple comparisons test was used for post hoc analysis. *p < 0.05.

To further determine the function of LINC00511 in chondrocyte pyroptosis, LINC00511 was silenced in LPS-induced ATDC5 cells (*p* < 0.05) (Fig. 2A). Subsequently, the role of LINC00511 knockdown in LPS-induced chondrocyte pyroptosis was examined, and the LPS-si-LINC00511 group exhibited reduced levels of IL-1β and IL-18 (*p* < 0.05) (Fig. 2B and 2C) and decreased levels of NLRP3, ASC, Caspase-1 and GSDMD (*p* < 0.05) (Fig. 2D and 2E). Moreover, a dual-luciferase reporter gene assay revealed that the LPS-si-LINC00511 group exhibited decreased caspase-1 fluorescence (*p* < 0.05) (Fig. 2F). In addition, LINC00511 knockdown increased chondrocyte viability (*p* < 0.05) (Fig. 2G) and decreased the LDH release rate (*p* < 0.05) (Fig. 2H). Thus, LINC00511 knockdown inhibited chondrocyte pyroptosis mediated by the NLRP3/Caspase-1 axis.

### LINC00511 Sponges miR-9-5p in LPS-Induced Chondrocyte Pyroptosis

**Fig. 3:**
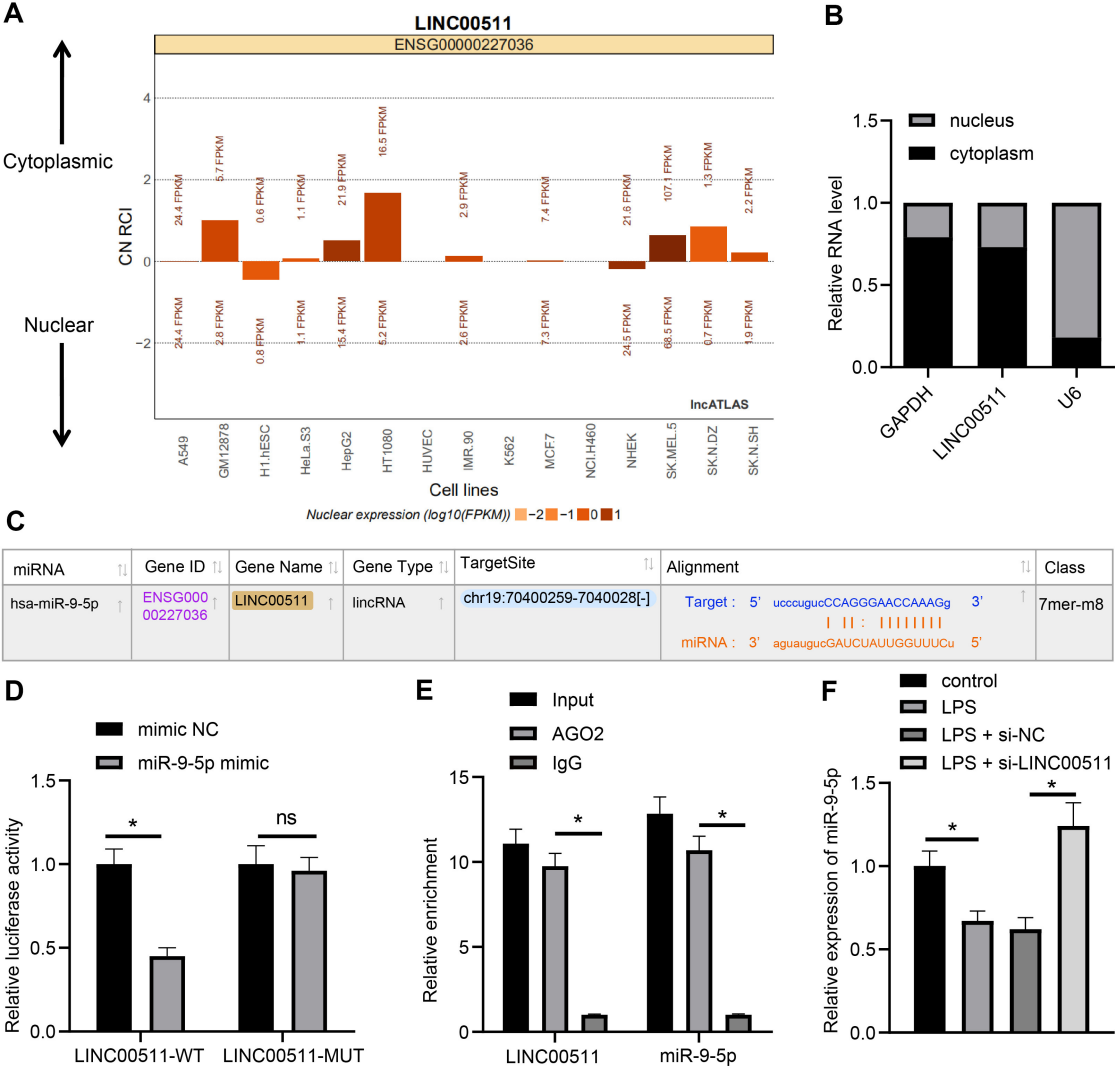
LINC00511 sponges miR-9-5p in LPS-induced chondrocytes. (**A**) LINC00511 subcellular localization predicted via the LncATLAS website (http://lncatlas.crg.eu/). (**B**) LINC00511 subcellular localization detected via fractionation of nuclear and cytoplasmic RNA. (**C**) The binding site between LINC00511 and miR-9-5p was predicted through the starBase website (http://starbase.sysu.edu.cn/index.php). D and E, Relationships between LINC00511 and miR-9-5p detected by dual-luciferase reporter gene assays (**D**) and RIP assays (**E**). (**F**) miR-9-5p expression in chondrocytes was measured by qRT‒PCR. Three independent repeated tests were conducted. The results are presented as the mean ± standard deviation. One-way ANOVA was used to analyse the data in Panel F. Two-way ANOVA was used to analyse the data in Panels B, D, and E. Tukey's multiple comparisons test was applied for the post hoc test. *p < 0.05.

To determine the mechanism of LINC00511 in chondrocyte pyroptosis, LINC00511 subcellular localization was predicted via the LncATLAS website (http://lncatlas.crg.eu/), which showed that LINC00511 was principally located in the cytoplasm (Fig. 3A), and it was mainly expressed in the cytoplasm of chondrocytes, as shown by fractionation of nuclear and cytoplasmic RNA (Fig. 3B), indicating that LINC00511 might modulate OA via the ceRNA network. Additionally, the starBase website (http://starbase.sysu.edu.cn/index.php) predicted that LINC00511 could bind to miR-9-5p (Fig. 3C). Furthermore, according to the results of the dual-luciferase reporter gene assay (*p* < 0.05) (Fig. 3D) and RIP assay (*p* < 0.05) (Fig. 3E), LINC00511 could sponge miR-9-5p. In addition, miR-9-5p expression in chondrocytes from each group was examined by qRT‒PCR, which revealed that compared with the control group, the LPS group exhibited downregulated miR-9-5p expression, while the LPS-si-LINC00511 group exhibited upregulated miR-9-5p expression (*p* < 0.05) (Fig. 3F). The above findings revealed that LINC00511 could sponge miR-9-5p to reduce miR-9-5p expression in LPS-induced chondrocytes.

### miR-9-5p Knockdown Neutralizes the Inhibitory Effect of LINC00511 Knockdown on LPS-Induced Chondrocyte Pyroptosis

**Fig. 4:**
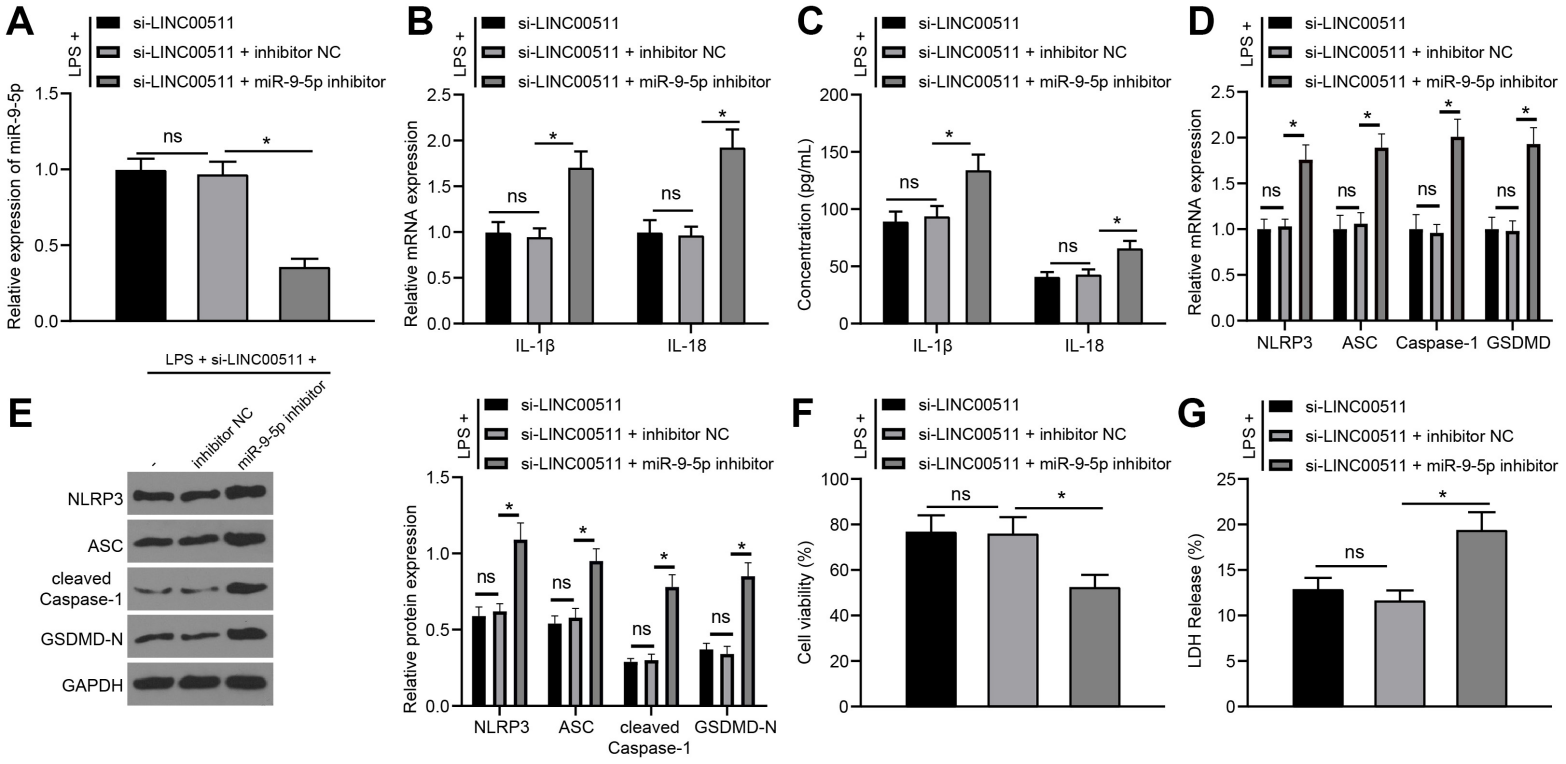
miR-9-5p underexpression neutralizes the inhibitory effect of LINC00511 knockdown on LPSinduced chondrocyte pyroptosis. LINC00511 and miR-9-5p were both silenced in LPS-treated ATDC5 cells. (**A**) miR-9-5p expression was examined via qRT‒PCR. B and C, Levels of IL-1β and IL-18 determined by qRT‒PCR (**B**) and ELISA (**C**). D and E, The levels of NLRP3, ASC, Caspase-1, and GSDMD were detected via qRT‒PCR (**D**) and western blot analysis (**E**). (**F**) Cell viability was tested with Cell Titer Glo Luminescent Cell Viability Assay kits. (**G**) LDH viability measurement. Three independent repeated tests were conducted. The results are presented as the mean ± standard deviation. One-way ANOVA was used to analyse the data in Panels A, F, and G. Two-way ANOVA was used to analyse the data in Panels B, C, D, and E. Tukey's multiple comparisons test was used for post hoc analysis. *p < 0.05.

Subsequently, functional rescue assays were conducted to validate the effect of simultaneous silencing of both LINC00511 and miR-9-5p in LPS-treated ATDC5 cells. First, miR-9-5p expression was tested by qRT‒PCR, which revealed that the si-LINC00511+miR-9-5p inhibitor group had lower miR-9-5p expression than the si-LINC00511+inhibitor NC group (*p* < 0.05) (Fig. 4A); however, the si-LINC00511 group and the si-LINC00511+inhibitor NC group showed no significant differences. The detection of chondrocyte pyroptosis revealed that the si-LINC00511+miR-9-5p inhibitor group had increased levels of IL-1β and IL-18 (*p* < 0.05) (Fig. 4B and 4C) as well as elevated expression of NLRP3, ASC, Caspase-1 and GSDMD (*p* < 0.05) (Fig. 4D and 4E) compared with the si-LINC00511+inhibitor NC group. Moreover, compared with the si-LINC00511+inhibitor NC group, the si-LINC00511+miR-9-5p inhibitor group exhibited reduced cell viability (*p* < 0.05) (Fig. 4F) and increased LDH release (*p* < 0.05) (Fig. 4G), while the si-LINC00511 group and the si-LINC00511+inhibitor NC group exhibited no significant differences. These findings suggested that LINC00511 knockdown could sponge miR-9-5p expression to suppress LPS-induced chondrocyte pyroptosis.

### LINC00511 Knockdown Sponges miR-9-5p to Quench FUT1 Expression in LPS-Induced Chondrocyte Pyroptosis

**Fig. 5:**
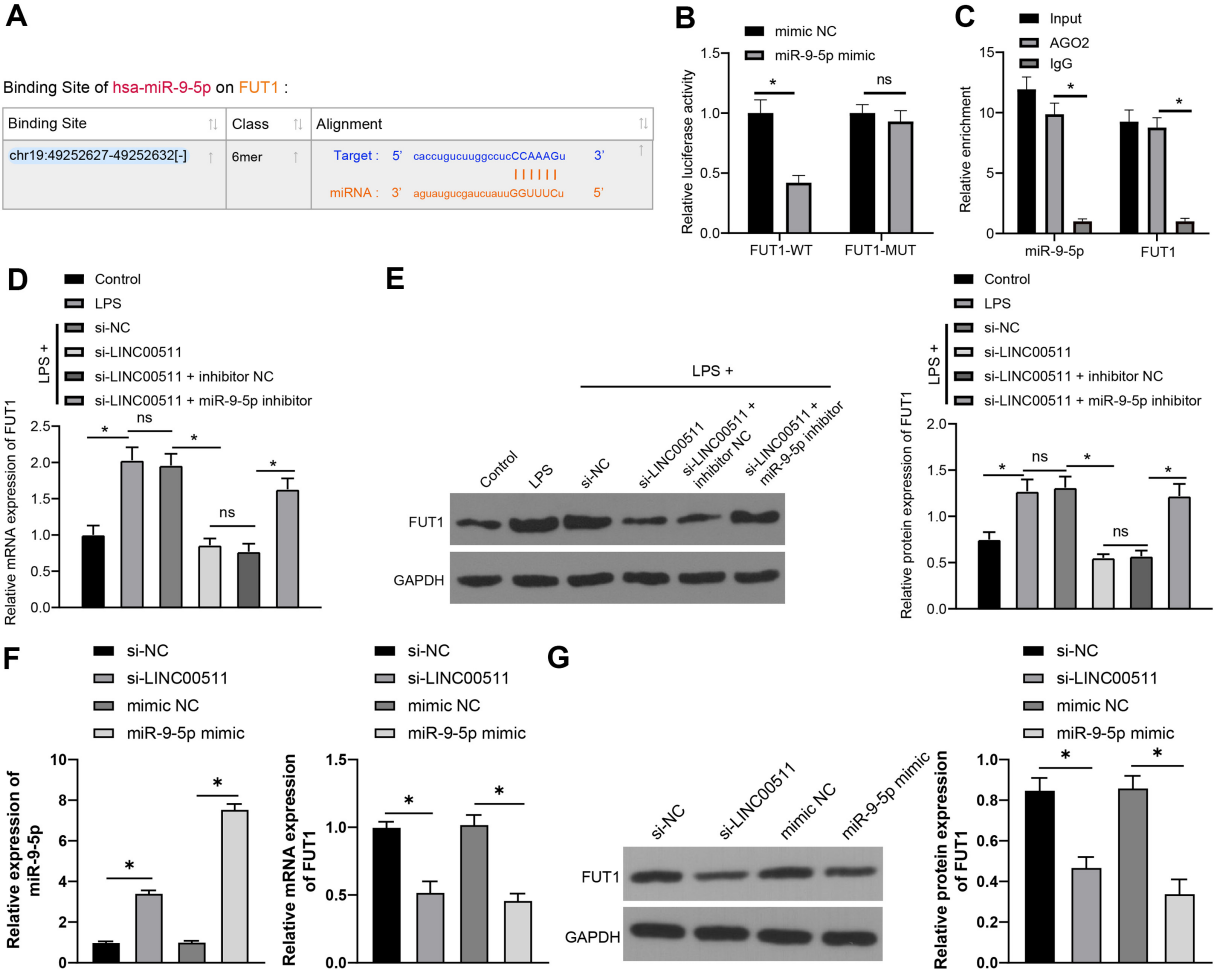
LINC00511 knockdown sponges miR-9-5p to quench FUT1 expression in LPS-induced chondrocyte pyroptosis. (**A**) The binding relationship between miR-9-5p and FUT1 was predicted by the starBase website (http:// starbase.sysu.edu.cn/index.php). (**B**) and (**C**) The binding relationship between miR-9-5p and FUT1 was verified by dualluciferase reporter gene assays (**B**) and RIP assays (**C**). (**D**) and (**F**) Levels of FUT1 or miR-9-5p determined by qRT‒PCR. E and G, Levels of FUT1 determined by Western blot analysis (**E, G**). Three independent repeated tests were conducted. The results are presented as the mean ± standard deviation. One-way ANOVA was used to analyse the data in Panels D and E. Two-way ANOVA was used to analyse the data in Panels B and C. Tukey's multiple comparisons test was applied for the post hoc test. *p < 0.05.

To clarify the downstream mechanism of miR-9-5p, the starBase website (http://starbase.sysu.edu.cn/index.php) predicted that miR-9-5p binds to FUT1 at the 3’ untranslated region (Fig. 5A). Dual-luciferase reporter gene assays (*p* < 0.05) (Fig. 5B) and RIP assays (*p* < 0.05) (Fig. 5C) revealed that FUT1 could serve as a direct target gene of miR-9-5p. Compared with that in the control group, FUT1 expression was increased in the LPS group, decreased in the si-LINC00511 group, and reversed in the si-LINC00511+miR-9-5p inhibitor group (*p* < 0.05) (Fig. 5D and 5E). In addition, we assayed the expression of FUT1 in chondrocytes after transfection with si-LINC00511 or the miR-9-5p mimic alone. Inhibition of LINC00511 or overexpression of miR-9-5p both significantly reduced intracellular FUT1 expression (*p* < 0.05, Fig. 5F and 5G). These results verified that LINC00511 knockdown sponged miR-9-5p to limit FUT1 expression in LPS-induced chondrocyte pyroptosis.

### FUT1 Overexpression Reverses the Inhibitory Effect of LINC00511 Knockdown on LPS-Induced Chondrocyte Pyroptosis

**Fig. 6:**
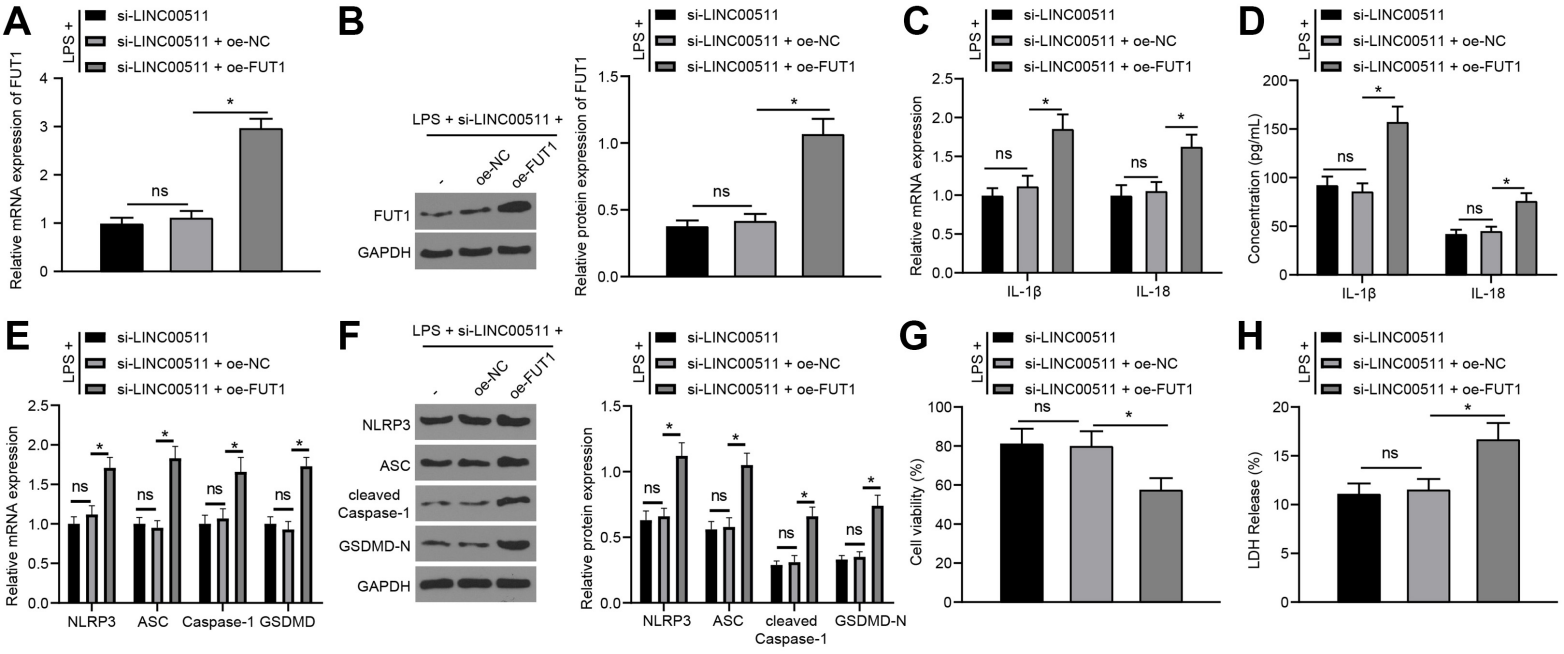
FUT1 overexpression reverses the inhibitory effect of LINC00511 knockdown on LPS-induced chondrocyte pyroptosis. LINC00511 was silenced while FUT1 was overexpressed in LPS-treated ATDC5 cells. A and B, FUT1 expression measured by qRT‒PCR (**A**) and western blot analysis (**B**). C and D, Levels of IL-1β and IL-18 verified by qRT‒PCR (**C**) and ELISA (**D**). E and F, The levels of NLRP3, ASC, Caspase-1, and GSDMD were detected via qRT‒PCR (**E**) and western blot analysis (**F**). (**G**) Cell viability was analysed by Cell Titer Glo Luminescent Cell Viability Assay kits. (**H**) LDH viability measurement. Three independent repeated tests were conducted. The results are presented as the mean ± standard deviation. One-way ANOVA was used to analyse the data in Panels A, B, G, and H. Two-way ANOVA was used to analyse the data in Panels C, D, E, and F. Tukey's multiple comparisons test was used for post hoc analysis. *p < 0.05.

To further verify that LINC00511 affects chondrocyte pyroptosis by modulating FUT1, LINC00511 was silenced, while FUT1 was overexpressed in LPS-treated ATDC5 cells. FUT1 expression was measured by qRT‒PCR and western blot analysis, which revealed that compared with that in the si-LINC00511+oe-NC group, FUT1 was strongly expressed in the si-LINC00511+oe-FUT1 group (*p* < 0.05) (Fig. 6A and 6B), while no significant differences were detected between the si-LINC00511 group and the si-LINC00511+oe-NC group. Next, compared with the si-LINC00511+oe-NC group, the si-LINC00511+oe-FUT1 group exhibited increased levels of IL-1β and IL-18 as well as increased levels of NLRP3, ASC, Caspase-1, and GSDMD (*p* < 0.05) (Fig. 6C-6F), reduced cell viability (*p* < 0.05) (Fig. 6G) and increased LDH release (*p* < 0.05) (Fig. 6H). Overall, LINC00511 knockdown reduced FUT1 expression to inhibit LPS-induced chondrocyte pyroptosis.

## Discussion

OA is a serious cartilaginous disease that results from an integrated number of systemic and regional causes and leads to chondrocyte damage and pyroptosis [24]. Pyroptosis results from the excessive release of inflammasomes and is characterized by leakage of GSDMD and an inflammatory response to cell death [25]. LncRNAs are indispensably involved in OA development [20]. LINC00511 overexpression is prevalent in a wide range of carcinomas and is responsible for advanced disease stage, tumour growth, lymph node metastasis, poor prognosis, a disappointing overall survival rate, and an elevated rate of relapse [26]. Through the collected evidence, it was quite clear that the studies on LINC00511 were mainly focused on multiple cancers. In this study, we investigated its role in chondrocyte pyroptosis in OA.

In our study, LPS activated the NLRP3 inflammasome to induce chondrocyte pyroptosis and upregulate LINC00511 expression via increased levels of IL-1β and IL-18, upregulated levels of NLRP3, ASC, Caspase-1 and GSDMD, and increased LDH release. When rats with OA were treated with anti-inflammatory and anti-osteoporosis drugs, caspase-1-dependent pyroptosis induced by the NLRP3 inflammasome was attenuated, suggesting the pernicious role of pyroptosis in OA [27]. OA chondrocyte inflammatory injury was related to progressive pyroptosis and the upregulation of inflammatory cytokine levels [5]. Recently, the NLRP3 inflammasome was shown to act as a potent driver of OA severity and pyroptosis, and it was shown to be positively correlated with inflammatory injury, ASC, Caspase-1, and GSDMD [1]. Moreover, the restriction of LDH release exerted a protective effect on chondrocytes in OA [28]. LINC00511 downregulation inhibited the NLRP3 inflammasome to reduce LPS-induced chondrocyte pyroptosis. Notably, LINC00511 is strongly expressed in OA, and sabotages chondrocyte dissemination and accelerates apoptosis [12]. Although the role of LINC00511 in pyroptosis or inflammation has yet to be elucidated, several studies have shown that LINC00511 overexpression is present in a substantial number of cancers, including bladder cancer, cervical cancer, and hepatocellular carcinoma, and promotes cancer cell growth, impedes apoptosis, enhances lymph node metastasis and results in an unfavourable overall survival rate [29, 30]. Therefore, LINC00511 serves as a dangerous biomarker in human diseases.

Mechanistically, LINC00511 could sponge miR-9-5p. LINC00511 suppressed miR-765 to facilitate osteosarcoma cell development, expansion, and colony formation [32]. Furthermore, LINC00511 could sponge miR-150-5p to increase oesophageal cancer viability and growth and block cancer cell death [33]. Overall, LINC00511 could function as a sponge of miRs to augment malignancies. On the other hand, miR-9-5p is involved in diabetes nephropathy progression by connecting cancer susceptibility candidate 2 and peroxisome proliferator-activated receptor γ to a ceRNA network [34]. Moreover, in chronic obstructive pulmonary disease, when miR-9-5p is sponged by RP11-86H7.1, its target is accordingly activated; thus, inflammatory responses are enhanced in the airway [35]. Moreover, the knockdown of miR-9-5p reversed the suppressive effect of LINC00511 silencing on LPS-induced chondrocyte pyroptosis. miR-9-5p contributes to preventing inflammatory damage and sustaining cartilaginous functions in OA by relieving oxidative stress and reducing inflammatory responses, indicating that miR-9-5p is conducive to alleviating OA [36]. It has been reported that miR-9-5p can mitigate diabetes by downregulating pyroptosis, which is coupled with reductions in IL-1β, IL-18, NLRP3, and Caspase-1 [37]. Moreover, miR-9-5p functions as a pivotal suppressor in cardiovascular diseases because it protects the vascular system from proinflammatory injury [38]. In a recent study, miR-9-5p was found to attenuate apoptosis and LDH release to relieve endoplasmic reticulum damage [39]. Taken together, these findings indicate that miR-9-5p might alleviate OA in an anti-inflammatory manner.

Importantly, silencing LINC00511 downregulated FUT1 by sponging miR-9-5p. As the downstream gene of the ceRNA interaction, FUT1 is controlled by ADP-dependent glucokinase antisense RNA 1 and miR-525 to affect colorectal cancer metastasis and malignancy [40]. In addition, as the target of miR-140-5p/149, FUT1 was inactivated to facilitate chondrocyte development and self-renewal and to quench cell apoptosis in OA [41]. Overall, FUT1 overexpression neutralized the inhibitory effect of LINC00511 silencing on LPS-induced chondrocyte pyroptosis. Amin *et al*. reported that FUT1 depletion decreased angiogenesis and leucocyte content in the inflammatory joints of patients with arthritis [42]. A previous study revealed that FUT1 was robustly expressed and responsible for inflammatory injury in the synovial tissues of rheumatoid arthritis patients [43]. In addition, the ceRNA interaction of LINC00511/miR-9-5p/FUT1 strongly participated in chondrocyte pyroptosis in OA.

Our study suggested that LINC00511 silencing inhibited the NLRP3 inflammasome to quench chondrocyte pyroptosis in OA by promoting miR-9-5p and downregulating FUT1. These results provide a novel theoretical strategy for OA treatment. However, we failed to further probe the upstream mechanism of aberrantly expressed LINC00511. We will attempt to probe the underlying mechanism of other targets of LINC00511 in OA. Microarray analysis of LPS-treated cells was not performed in our experiments. Therefore, there may be other lncRNAs with more significant changes in expression levels than LINC00511, or there may be other lncRNAs involved in chondrocyte pyroptosis. Therefore, more exploration is needed. More efforts will be made to identify curative targets of OA. Although these findings offer possible strategies for OA treatment, preclinical research is needed, and the experimental outcomes and practical application in clinical practice need intensive investigation.

## Figures and Tables

**Table 1:** Primer sequences for qRT‒PCR.

	Forward Primer (5'-3')	Reverse Primer (5'-3')
LINC00511	CGGCTTAACTAACTGTTACTC	CAGTACCGATGTCAGACACGGA
miR-9-5p	GGAGTCCGTGTGTCTGTGTG	GCTTTATGA CGGCTCTGTGG
FUT1	AAAGCGGACTGTGGATCT	GGACACAGGATCGACAGG
IL-1β	CTTCAGGCAGGCAGTATCACTC	TGCAGTTGTCTAATGGGAACGT
IL-18	GCCTCAAACCTTCCAAATCA	TGGATCCATTTCCTCAAAGG
NLRP3	AGCCTTCCAGGATCCTCTTC	CTTGGGCAGCAGTTTCTTTC
ASC	GACAGTGCAACTGCGAGAAG	CGACTCCAGATAGTAGCTGACAA
Caspase-1	ACACGTCTTGCCCTCATTATCT	ATAACCTTGGGCTTGTCTTTCA
GSDMD	CCAACATCTCAGGGCCCCAT	TGGCAAGTTTCTGCCCTGGA
U6	CTCGCTTCGGCAGCACA	AACGCTTCACGAATTTGCGT
GAPDH	CAGTCACTACTCAGCTGCCA	GAGGGTGCTCC GGTAG

qRT‒PCR, quantitative real-time polymerase chain reaction; LINC, long intergenic nonprotein coding RNA; miR, microRNA; FUT1, fucosyltransferase 1; IL, interleukin; NLRP3, nod-like receptor pyrin domain 3; ASC, apoptosis-associated speck-like protein; GSDMD, gasdermin D, GAPDH, glyceraldehyde-3-phosphate dehydrogenase
